# Suicide deaths among reproductive-aged women in the US post-*Dobbs*: a national time-series analysis

**DOI:** 10.1007/s00127-025-02902-7

**Published:** 2025-04-17

**Authors:** Parvati Singh, Alaxandria Crawford, Sarah Crow, Jonathan R. Powell, Maria F. Gallo

**Affiliations:** 1 Division of Epidemiology, College of Public Health, The Ohio State University, 338 Cunz Hall, 1841 Neil Avenue, Columbus, OH 43210 USA; 2 National Registry of Emergency Medical Technicians, Columbus, USA; 3 Department of Epidemiology, Gillings School of Global Public Health, University of North Carolina, Chapel Hill, USA

**Keywords:** *Dobbs*, Suicides, Abortion restriction, Time-series analysis

## Abstract

**Purpose:**

The United States Supreme Court’s *Dobbs* decision in June 2022 may have worsened mental health among reproductive-aged women nationally. We examined whether the *Dobbs* decision preceded an increase in suicides among reproductive-aged women using national, monthly data, from January 2018-December 2023.

**Methods:**

We retrieved national monthly suicide counts from January 2018 to December 2023 for women and men 15–49 years of age (overall and stratified by two age groups- 15–24 years, 25–49 years) from the Centers for Disease Control and Prevention’s Wide-ranging Online Data for Epidemiologic Research Multiple Cause of Death database. We used time series analyses to examine whether residuals of nationally aggregated counts of monthly suicides among women 15–49, 15–24- and 25–49-years of age (outcomes) exhibited higher-than-expected values following the *Dobbs* decision, controlling for autocorrelation and concomitant monthly series of suicides among men.

**Results:**

We observed higher-than-expected residuals of suicides in July and September 2022 among 15–49-year-old women, and in September, October, December 2022 and March 2023 among 15–24-year-old women. No residual outliers were observed among 25–49-year-old women post-*Dobbs*. Results from time-series analyses indicate an average of 52.5 additional suicides in outlier months among 15–49-year-old women post-*Dobbs* (95% confidence interval [CI]: 14.85, 90.15). The increase appeared pronounced among younger age (15–24 years) women (coefficient = 19.6, 95% CI: 11.17, 28.03). Results suggest 104 additional suicides among 15–49-year-old women, and 78 excess suicides among 15–24-year-old women, nationally, post-*Dobbs*.

**Conclusions:**

Findings highlight the adverse impact of the *Dobbs* ruling on mental health among reproductive-aged women.

**Supplementary Information:**

The online version contains supplementary material available at 10.1007/s00127-025-02902-7.

## Introduction

The U.S. Supreme Court’s *Dobbs v. Jackson Women’s Health Organization* decision in June 2022 overturned the federal right to abortion previously established by *Roe v. Wade* (1973). The *Dobbs* ruling returned regulatory authority over abortion to states. Subsequently, as of August 2024, 22 states have banned or heavily restricted abortion, whereas others have maintained or improved access [1]. The ruling has raised grave concerns regarding the physical, psychological, and social implications of restricted access to abortion care [2–6]. Unsurprisingly, the immediate *post-Dobbs* period has been marked by higher mental distress, anxiety and depression symptoms among reproductive-aged women [7–9].

Restricting access to abortion care may harm mental health among people with the capacity for pregnancy, regardless of their pregnancy status [2, 3, 7–13]. Abortion restrictions may induce a cascade of stressors such as loss of autonomy, stigma, interpersonal conflict, and socio-economic challenges that may worsen mental health in this population [2, 3, 7–13]. Importantly, abortion itself does not cause adverse mental health; rather, the denial of choice may lead to significant distress, which is an important distinction for understanding the mental health effects of restrictive abortion laws [14–16].

Pregnancy intention forms a key predictor of mental health among pregnant people [12]. People with unintended pregnancies exhibit nearly twice the risk of depression relative to their counterparts with intended pregnancies, with symptoms often persisting beyond pregnancy [17, 18]. Individuals with pre-existing mental health conditions or those who experience traumatic events, such as intimate partner violence, may face more severe outcomes if denied abortion care [10, 11]. Anxiety, depression, trauma, and exposure to violence and abuse may increase the risk of suicide mortality. An ecological study examining the association between abortion restrictions and suicides in the US found a 6% increase in state-level suicides among reproductive-aged women following enforcement of restrictive abortion policies, from 1974 to 2016[12]. Denial of abortion services and associated stigma are linked to lasting mental distress, extending well beyond pregnancy. This mental distress may negatively affect self-esteem and life satisfaction, potentially eroding optimism about the future or positive future orientation [19–21].

Positive future orientation, or an individual’s optimistic outlook on their potential future, is closely linked to lower suicide risk [22–27]. When people envision a hopeful future, they are more likely to feel motivated to overcome present challenges and view their lives as meaningful. This sense of purpose and hope serves as a protective factor against suicidal thoughts and behaviors [28, 29]. Research consistently shows that individuals with a strong belief in their ability to achieve future goals and aspirations experience lower levels of depression and hopelessness, which are two key predictors of suicide [22–27]. Conversely, a lack of positive future orientation, characterized by pessimism or a sense of hopelessness about what lies ahead, increases the risk of suicidal ideation, self-harm and suicide mortality [22–27]. This relation is also observed at the population level, with decline in collective optimism about the future corresponding with higher-than-expected suicide mortality [30, 31]. Abortion restrictions can exacerbate this dynamic, particularly for reproductive-aged women, as they may face significant life disruptions, including financial instability, loss of autonomy, and social stigma [19–21]. These stressors can diminish a person’s sense of control over their future and foster a pessimistic outlook, increasing mental distress. Policies limiting reproductive autonomy, such as the *Dobbs* decision, may thus elevate suicide risk by undermining hope and optimism for the future across the population.

In this study, we examine whether and to what extent, the timing of the *Dobbs* decision (June 2022) precedes an increase in suicides among reproductive-aged women (15–49 years) nationally in the US. We hypothesize that the *Dobbs* ruling (June 2022) would precede an immediate increase in suicides among reproductive-age women (15–49 years) nationally in the U.S. Young women exhibit the highest risk of unintended pregnancy and pregnancy-related adverse mental health symptoms in the US [32, 33]. As such, we also hypothesize that the post-*Dobbs* increase in suicides would be higher among young (15–24 years) reproductive-age women relative to their older counterparts (25–49 years).

## Methods

### Data

We retrieved national monthly data on suicide deaths for reproductive-aged (15–49 years) women and similarly-aged men from January 2018 to December 2023 (72 months), from the Centers for Disease Control and Prevention’s (CDC) Wide-ranging Online Data for Epidemiologic Research (WONDER) Multiple Cause of Death database[34]. We further stratified suicide data for women and men by two age groups: 15–24 years and 25–49 years. CDC WONDER is a publicly accessible system that provides cause of death-specific mortality data for the US, with provisional aggregate data available for recent months and years. We identified suicide deaths using International Classification of Diseases (ICD) codes (X60-X84), by month, gender (male/female) and age groups (15–24 vs. 25–49 y) across underlying causes and contributing factors (i.e., multiple causes) compiled by the CDC using death certificate databases. We opted for these two broad age groups based on (i) age-group-specific risk of unintended pregnancy [32, 33], and (ii) changes in abortion rates immediately preceding 2022 in the US wherein abortion rates from 2020 to 2021 increased among women aged 15–24 years, but declined/did not change among women aged 25 years and above [35].

### Variables

We defined, as our outcomes, the monthly count of suicides nationally in the US (2018–2023) for reproductive-aged women overall and for the two age subgroups: 15–24 years and 25–49 years. We utilized the concomitant series of monthly suicides among men (per age group) as a control variable. We defined our exposure as the timing of the *Dobbs* decision (June 2022).

### Analysis

The monthly patterning of population-level suicides may exhibit seasonality, trends and temporal patterns, collectively referred to as autocorrelation [36]. Autocorrelation violates fundamental requirements of correlational tests as the observed values of any monthly series of suicides in a stable population are not independent of prior values and may exhibit non-constant variance. AutoRegressive Integrated Moving Average (ARIMA) time-series analysis offers an efficient way to address these violations and is suitable for examining the association of exogenous exposures (e.g., the *Dobbs* decision) with suicides due to its ability to model complex temporal data and account for trends, seasonality, and random fluctuations [37, 38]. ARIMA’s autoregressive (AR) component captures the relationship between current suicide counts and past observations, allowing us to control for and remove the effect of past levels of suicides on future patterns [37, 38]. The moving average (MA) component models the residuals or “shocks” from previous time points, accounting for irregular fluctuations in suicides following the *Dobbs* decision [37, 38]. Suicide data may exhibit non-stationarity, where patterns like trends or seasonality change over time. ARIMA models are designed to address non-stationarity through differencing (the “integrated” component), which removes secular trends and stabilizes the mean [37, 38]. Taken together, ARIMA analysis helps in identifying the core signal of a series using the AR, I, MA parameters (referred to as the ARIMA “signature” of a series) that collectively yield the unobserved counterfactual (i.e. fitted or predicted values) and residual values (observed less fitted values) [37–40]. ARIMA-derived residuals offer analytic benefits of zero mean and constant variance, and permit examination of the association between an exogenous exposure and consequent perturbations in the outcome, net of autocorrelation [37–40]. For this longitudinal, observational, ecologic study, we used ARIMA analysis to examine the association between the timing of the *Dobbs* decision and ARIMA-derived residuals of observed series of monthly suicides among reproductive-aged women in the US through the following 4 steps using Scientific Computing Associates software designed for time series analyses [41].

1. We used Box-Jenkins iterative pattern recognition routines to identify the ARIMA signature of our outcome series in the pre-*Dobbs* period (i.e., January 2018-May 2022), controlling for the concomitant series of suicides among men of the same age group. This control series (also referred to as a covariate transfer function) helped account for shared temporal patterning of suicides across women and men over our study period, including any shared influence of other macrosocial stressors (e.g., the COVID-19 pandemic).
2. We used the pre-*Dobbs* ARIMA signature identified in step 1 to forecast (predict) values of the outcome for the 18-month period after the *Dobbs* decision (i.e. June 2022 to December 2023). These predicted or fitted values served as statistical counterfactuals and reflected the expected monthly count of female suicides if, counter to fact, the *Dobbs* decision had not occurred [39, 40].
3. We used the ARIMA signature identified in step 1 and expected values from step 2 to obtain residual values (observed less fitted) of our outcome series.
4. We used the pre-*Dobbs* standard error of residuals to develop pre-*Dobbs* 95% confidence intervals (CI; prediction interval of monthly series of suicides among reproductive women overall and for the two age groups from January 2018 to May 2022). We extended the pre-*Dobbs* prediction interval to the full residual series (January 2018-December 2023) for each outcome. Next, we noted whether any residuals deviated from this prediction interval post-*Dobbs* (i.e., June 2022 onward) [42–48].
5. If the outcome series exhibited outliers in the post-*Dobbs* period following step 4, we created a binary indicator of the month-specific timing of any identified outliers in residuals of suicides among reproductive-aged women post-*Dobbs* (1 for months with outliers, 0 otherwise) to quantify the average change in suicides post-*Dobbs*. This data-driven approach also helps avoid multiple testing that could increase chances of false rejection of the null.
6. We applied the binary indicator of month-specific outliers (from step 5) to the ARIMA-derived residuals of outcomes to determine the magnitude of outcome deviation from expected levels in the months with residual outliers. This sequential approach (steps 1–6) has also been utilized in prior research examining changes in reproductive health outcomes following unprecedented exogenous macrosocial shocks, particularly in circumstances where exposure lags are difficult to hypothesize *a priori* [42–48].

Owing to our use of publicly available, aggregate, de-identified data, this study was deemed exempt from review by The Ohio State University’s Institutional Review Board. We followed the Strengthening the Reporting of Observational Studies in Epidemiology (STROBE) guidelines for reporting observational studies. We specified 2-tailed tests using statistical significance level of p < 0.05.

## Results

**Fig. 1:**
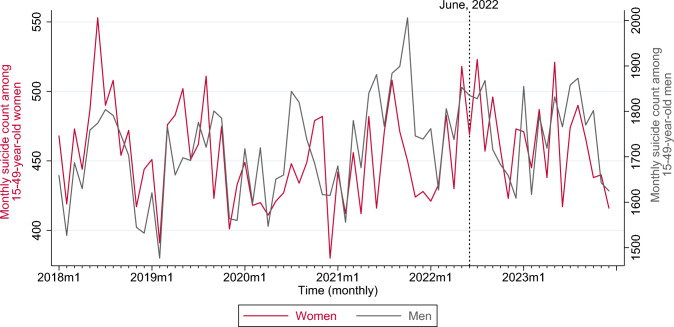
Monthly trends in suicides (count) among women (red) and men (gray) 15–49 years of age, United States, 2018–2023

Our analytic dataset (2018–2023, 72 months) comprised 156,847 suicides among women and men 15–49 years of age, of which about 20.9% occurred among women (Table 1). Over our study period, suicide counts averaged 455.1 per month among reproductive-aged women, and were markedly lower compared to the mean count among men (1723.4), in congruence with other reports (Table 1) [49]. Nationally, monthly suicides averaged 101.5 among younger reproductive-aged (15–24 years) women, and 353.6 among older, reproductive-aged (25–49 years) women. Figure 1 graphs the trend in monthly suicides among reproductive-aged women and men over our study period. Supplement Figure S1 shows national monthly suicide trends among women in the younger and older age groups.

**Table 1:** Suicides among women and men by age groups, United States, 2018–2023

	Total count	Monthly mean	Monthly Standard Deviation
Women 15–49 years	32,765	455.1	34.4
15–24 years	7307	101.5	12.9
25–49 years	25,458	353.6	31.4
Men 15–49 years	124,082	1723.4	105.9
15–24 years	29,440	408.9	32.3
25–49 years	94,642	1314.5	90.7

*SD* standard deviation

**Fig. 2:**
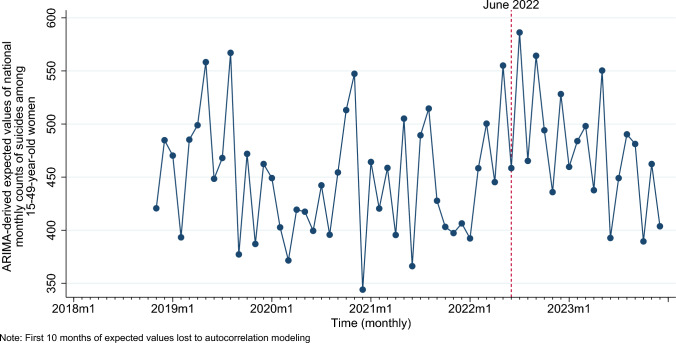
Time-series graph of ARIMA-derived expected (predicted) values of suicides among women 15–49 years of age, United States, January 2018 to December 2023

Over the pre-*Dobbs* period (January 2018- May 2022), Box-Jenkins routines identified AR(10) parameter as the ARIMA signature of the national monthly series of suicides among reproductive-aged women, controlling for the concomitant series of male suicides (Table 2). The residuals over the pre-*Dobbs* period exhibited a standard deviation of 29.1 (Table 2), which was used to develop a 95% Confidence Interval (i.e. pre-*Dobbs* prediction interval) of outcome residuals. Predicted or expected values of the national monthly series of suicides (obtained from the pre-*Dobbs* ARIMA signature) for the full study period (January 2018 to December 2023) among reproductive-aged women are shown in Fig. 2. The difference between observed and expected values (observed less expected) yields the outcome residuals.

**Table 2:** Time-series parameters and Residuals statistics for pre-*Dobbs* identification of ARIMA signature for the series of national monthly suicides among women 15–49 years of age, United States, January 2018 to May 2022

Parameters	Coefficient	95% Confidence Interval	
		Lower bound	Upper bound
Suicides among 15–49-year-old males	0.25****	0.24	0.26
Autoregression (AR) lag			
10	0.44***	0.17	0.71
Residuals statistics			
T-value of residual mean (against zero)	0.29		
Standard deviation of residuals	29.1		

*p < 0.1; **p < 0.05, ***p < 0.01, ****p < 0.001; two-tailed test

*AR* autoregression; *CI* confidence interval

**Fig. 3:**
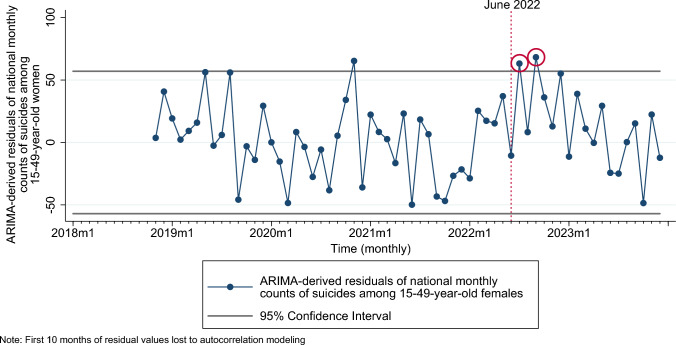
Time-series graph of ARIMA-derived residuals of suicides among women 15–49 years of age and pre-*Dobbs* 95% confidence (prediction) interval of residuals (applied to full series), United States, January 2018 to December 2023. Residual outliers post-*Dobbs* (June 2022) are circled in red

Application of pre-*Dobbs* prediction interval to ARIMA-derived residuals of suicides among reproductive-aged women for the full outcome series (January 2018 to December 2023) revealed higher-than-expected suicides in July and September 2022 (Fig. 3). Results from ARIMA time-series analyses showed an average of 52.5 additional suicides (95% CI: 14.85, 90.15) in these outlier months (coded as a composite binary variable indicating months with residual outliers post-*Dobbs*) among reproductive-aged women (Table 3). This increase corresponds with 104 additional suicides nationally, among reproductive-aged women immediately following the *Dobbs* decision (coefficient of binary outlier indicator × number of outlier months = 52 × 2 = 104).

**Table 3:** Results from ARIMA time-series analysis of monthly suicides among women 15–49 years of age, as a function of binary indicator of months with residual outliers post-*Dobbs*, concomitant series of age group-specific male suicides and autocorrelation, United States, 2018–2023

Variables	Coefficient	95% CI	
		Lower bound	Upper bound
Binary indicator of months with residual outliers post-*Dobbs*	52.5***	14.85	90.15
Suicides among men 14–49 years of age	0.26****	0.25	0.27
Autocorrelation parameters			
AR 10	0.33**	0.11	0.55

*p < 0.1; **p < 0.05, ***p < 0.01, ****p < 0.001; two-tailed test

*AR* autoregression, *CI* confidence interval

**Fig. 4:**
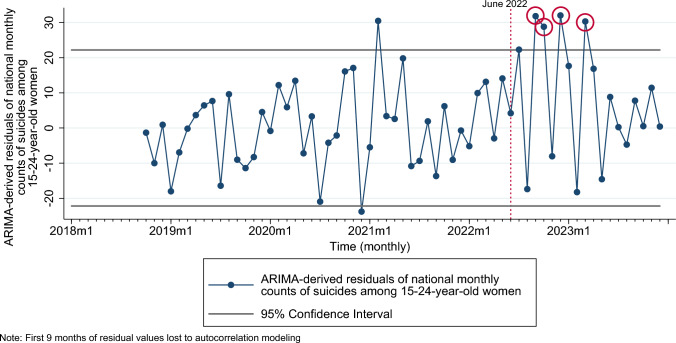
Time-series graph of ARIMA-derived residuals of suicides among women 15–24 years of age and pre-*Dobbs* 95% confidence (prediction) intervals of residuals (applied to full series), United States, January 2018 to December 2023. Residual outliers post-*Dobbs* (June 2022) are circled in red

The pre-*Dobbs* ARIMA parameters for suicides among 15–24-year and 25–49-year-old reproductive-aged women are presented in Supplement Table S1 and corresponding expected values are presented in Supplement Figure S2. Examination of the patterning of ARIMA-derived residuals indicates positive outliers in September, October, December 2022 and March 2023 among 15–24-year-old women (Fig. 4), but not among the 25–49-year-old group (Supplement Figure S3). Results from time-series analyses, with outlier months coded as a binary variable, suggest an average of 19.6 additional suicides (95% CI: 11.17, 28.03) among 15–24-year-old women over 4 outlier months that corresponds with 78.4 excess suicide deaths in this age group post-*Dobbs* (Table 4). We did not test this relation among 25–49-year-old women owing to no residual outliers observed post-*Dobbs* for this group (Supplement Figure S3).

**Table 4:** Results from ARIMA time-series analysis of monthly suicides among women 15–24 years of age, as a function of binary indicator of months with residual outliers post-*Dobbs*, concomitant series of age group-specific male suicides and autocorrelation, United States, 2018–2023

Variables	Coefficient	95% CI	
		Lower bound	Upper bound
Binary indicator of months with residual outliers post-*Dobbs*	19.6****	11.17	28.03
Suicides among men (per age group, respectively)	0.25****	0.25	0.25
Autocorrelation parameters			
AR 3	− 0.39***	− 0.64	− 0.14
AR 6	− 0.30**	− 0.56	− 0.04

*p < 0.1; **p < 0.05, ***p < 0.01, ****p < 0.001; two-tailed test

*AR* autoregression, *CI* confidence interval, NA not applicable

Across all our analyses, we do not observe any negative residual outliers in suicides among reproductive-aged women post-*Dobbs*. The autocorrelation function values and Ljung-Box Q test statistics for ARIMA models (pre-*Dobbs* identification of ARIMA signature) are presented in Supplement Table S2, which indicate absence of residual autocorrelation over first 12 lags for all specified models [50].

## Discussion

We found an increase in suicides among reproductive-aged women in the U.S. following the *Dobbs* decision, controlling for autocorrelation, secular trends, seasonality, and the possible effect of other contemporaneous macrosocial shocks. The increase was more pronounced among younger (15–24-year-old) women post-*Dobbs*. Our findings extend recent research documenting that reproductive-aged women experienced higher mental distress, anxiety and depression symptoms immediately post-*Dobbs *[7, 8]. Our focus on suicide deaths calls attention to the most serious of mental health conditions. Furthermore, by using mortality outcomes from vital records, which have the benefits of universal coverage and standardization in their documentation of data [51], our findings are less susceptible to misclassification or selection bias, which could have been present in earlier studies of mental conditions post-*Dobbs* that relied on self-reported survey data [7, 8]. It is also important to note that the effect of the *Dobbs* decision could extend beyond pregnant persons; its psychiatric impact could ripple across all persons with the capacity for pregnancy, regardless of current pregnancy status [7, 8].

Strengths of our analysis include the use of publicly available national data that permit independent verification and replication of our results. Our use of time-series analysis reduces confounding from secular trends, seasonality, and autocorrelation [37]. Our analytic approach establishes temporal order in that the *Dobbs* decision precedes changes in the outcome and our use of concomitant series of male suicides as covariate transfer functions reduces potential confounding from shared antecedents of changes in the population-level patterning of suicides. For our results to arise from an external national shock other than the timing of the *Dobbs* decision, such a factor would have to: (1) be unrelated with the *Dobbs* decision but occur in the period of June 2022, (2) correspond with an increase in suicides among 15–49 and 15–24-year-old women but not among men of the same age group, and (3) be uncorrelated with any downstream consequences of the *Dobbs* decision pertaining to mental health of women. We know of no such factor and contend that the timing of the *Dobbs* decision offers the most parsimonious explanation for our observed pattern of results.

As with any ecological analysis, limitations include that our results are not indicative of the individual-level risk of suicide following abortion restrictions [52]. We also cannot comment on whether the higher-than-expected count of suicide decedents identified in our results sought abortion care, had pre-existing psychiatric conditions, or were victimized through intimate partner violence following the *Dobbs* ruling. Our results, rather, pertain to a national psychiatric response indicated by the most extreme outcome of adverse mental health- suicide- following a large shock represented by the *Dobbs* decision. We encourage future research to examine the role of precipitating circumstances in suicidal ideation/self-harm and suicide mortality following restrictive abortion legislations using detailed healthcare and social services utilization data.

For this study, we relied on the CDC WONDER database, which categorizes suicide data into two gender categories: males and females. However, this binary classification fails to account for the mental health challenges experienced by minoritized sexual and gender groups, who may face disproportionately adverse health consequences following abortion restrictions [53]. These groups often encounter heightened stress, stigma, and barriers to reproductive [54, 55] and mental health care [56, 57], which may exacerbate the risk of suicide post-*Dobbs*. By only considering male and female data, we likely missed key nuances regarding the broader impact of abortion restrictions on diverse sexual and gender identities. We acknowledge this limitation and encourage future research to collect and analyze more inclusive identity data. This will be crucial to understanding the mental health outcomes of abortion restrictions, particularly among vulnerable and marginalized populations, and may help develop more inclusive health policies.

Our findings suggest that the *Dobbs* decision may have served as a national-level policy shock that preceded a surge in suicides among reproductive-aged women. We encourage future research to build upon the present study and extend our national-level analysis to detailed state-specific changes in suicides among reproductive-aged females following enactment of abortion restrictions. Owing to data restrictions within CDC WONDER (suppression of mortality data for cell sizes < 10), we were unable to examine monthly state-level changes in suicides post-*Dobbs*. Future research may utilize detailed restricted-use mortality vital statistics data to examine state-level changes in suicides among reproductive-aged women in relation to month-specific changes in abortion laws pre- and post-*Dobbs* [58]. Extant research uses broad state-group categorizations to examine differential impact of the *Dobbs* ruling on mental health symptoms among women in restrictive versus protective states. For instance, Thornburg et al. (2024) leverage trigger bans and report differentially higher adverse mental health symptoms among reproductive-aged women in states that enacted trigger bans post-*Dobbs*, relative to others [7]. Similarly, Dave et al. (2023) group states by trigger and/or anticipatory abortion bans and find higher mental distress among 18–44-year-old women in restrictive states within 3 months following the *Dobbs* decision [8]. However, anticipatory restrictions or trigger ban-based categorizations may not fully capture the complexities in state-based abortion laws that preceded and continued after *Dobbs*. Several states increased abortion restrictiveness after June 2022 by enacting new (non-trigger) laws (e.g., 6-week gestational bans) while others increased protections concomitantly (e.g., shield laws) [59]. On the other hand, some states enacted severe abortions restrictions several months prior to *Dobbs*. For instance, Texas enacted a near-total abortion ban almost one year before *Dobbs*, and it remains unclear if the state’s trigger ban following *Dobbs* substantially changed abortion access as the existing laws (pre-*Dobbs*) were already heavily restrictive [60, 61]. In other states, abortion restrictions increased over time post-*Dobbs*, despite the absence of trigger bans. The state of Florida, for instance, did not have a trigger ban in place but instituted increasingly harsher abortion restrictions between June 2022 and June 2024 [59]. Anderson et al. (2024) use the state-level timing of total or 6-week gestational bans to examine proximate changes in anxiety symptoms among women post-*Dobbs* [9]. However, this categorization does not account for states like Ohio which imposed a 6-week ban immediately after *Dobbs* [62] but later rescinded the ban following judicial directives [63]. More recently, several states voted to expand abortion access in November 2024, with some formerly restrictive states overturning restrictive abortion laws [64]. Owing to the rapidly evolving landscape of abortions laws, detailed analyses at the state-month level may aid our understanding of how abortion restrictions correspond with suicides. We encourage researchers to develop state-month-level abortion restrictiveness scales that would account for the spatial and temporal patterning of abortion laws in the present context. This type of spatio-temporal index is needed to examine changes in suicides among reproductive-aged women at sub-national levels.

Given prior research indicating that emergency department (ED) visits and inpatient admissions for self-harm and suicidal ideation closely track suicide mortality [65, 66], we encourage future research to examine whether these visits increased in response to abortion restrictions following the *Dobbs* decision. National and state level electronic health records or insurance claims-based datasets that provide high temporal and spatial resolution may be used to assess whether observed increases in suicide mortality among reproductive-aged women align with trends in suicide-related healthcare utilization. This approach may (i) independently verify the consistency of our findings with respect to the timing of population-level increase in suicide-related outcomes post-*Dobbs* and (ii) assess whether heightened suicide risk is reflected not only in mortality data but also in patterns of healthcare-seeking behavior for suicidal ideation/self-harm.

The provision of mental health care to those with the capacity for pregnancy, particularly younger people, is critical given that psychiatric comorbidities are one of the leading risk factors for maternal mortality [67–69]. Our observed national increase in suicides among women following the loss of the federal constitutional right to abortion may foreshadow a broader rise in maternal mortality, as untreated mental health issues are likely to escalate with increasing abortion restrictions. Anecdotal reports align with this concern, underscoring the urgent need for mental health services [70]. Additionally, *Dobbs* may portend grave implications for postpartum depression. People who were unable to access needed abortion services could face increased emotional and psychological distress, elevating their risk of postpartum depression and, consequently, suicide [71]. This highlights the need for integrating mental health care into reproductive health services, especially in contexts where reproductive options are restricted, to mitigate the potential for adverse outcomes.

## Conclusion

The *Dobbs* decision has fundamentally altered the reproductive rights landscape in the US. The denial of reproductive choice not only violates a human right to autonomy but may exert grave health, social, and economic consequences. While evidence is accumulating on the health harms from abortion restrictions, limited studies have examined the potential effects on mental health[2, 3, 7–13]. Our finding that suicide deaths increased among reproductive-age women following *Dobbs* indicates that more research is urgently needed in this area.

## Supplementary Information

Below is the link to the electronic supplementary material.

Supplementary file1 (DOCX 62 KB)

## Data Availability

Data availability statement: Data used in this study are publicly available at https://wonder.cdc.gov/.
