# Supplementary material for: Suicide deaths among reproductive-aged women in the US post-Dobbs: a national time-series analysis
Source: Soc Psychiatry Psychiatr Epidemiol. 2025 Apr 17;60(8):1835–45. doi: 10.1007/s00127-025-02902-7 (PMC12325476; doi:10.1007/s00127-025-02902-7)
Supplement: Supplementary file 1 — Supplementary file1 (DOCX 62 KB) [file 127_2025_2902_MOESM1_ESM.docx]

**Supplemental Material**

**Title: Suicide deaths among reproductive-aged women in the US post-*Dobbs*: A time-series analysis**

Parvati Singh, PhD^1^

Alaxandria Crawford, MPH^1^

Sarah Crow, MPH^1^,

Jonathan R. Powell, MPA^1,2^,

Maria F. Gallo, PhD^3^

^1^Division of Epidemiology, College of Public Health, The Ohio State University, USA

^2^National Registry of Emergency Medical Technicians, USA

^3^Department of Epidemiology, Gillings School of Global Public Health, University of North Carolina, USA

**Contents:**

1. Supplement Figure S1: Monthly trends in suicides (count) among women 15-24 years (red) and 25-49 years (gray), United States, 2018-2022
2. Supplement Table S1: Time-series parameters and Residuals statistics for pre-*Dobbs* identification of ARIMA signature for the series of monthly suicides among (i) 15-24-year-old women (Model a), and (ii) 25-49-year-old women (Model b), United States, January 2018 to May 2022.
3. Supplement Figure S2: Time-series graph of ARIMA-derived expected (predicted) values of suicides among women 15-24 years (red) and 25-49 years of age (gray), United States, January 2018 to December 2023.
4. Supplement Figure S3. Time-series graph of ARIMA-derived residuals of suicides among women 25-49 years of age and pre-*Dobbs* 95% confidence (prediction) intervals of residuals (applied to full series), United States, January 2018 to December 2023. Residual outliers post-*Dobbs* (June 2022) are circled in red.
5. Supplement Table S2: Results from Ljung-Box Q test of residual autocorrelation in the series of monthly suicides among (i) 15-49-year-old women (Model a), (ii) 15-24-year-old women (Model b), and (iii) 25-49-year-old women (Model c).

**Supplement Figure S1: Monthly trends in suicides (count) among women 15-24 years (red) and 25-49 years of age (gray), United States, 2018-2023**

**Supplement Table S1: Time-series parameters and Residuals statistics for pre-*Dobbs* identification of ARIMA signature for the series of monthly suicides among (i) 15-24-year-old women (Model a), and (ii) 25-49-year-old women (Model b), United States, January 2018 to May 2022.**

|  | **Model a: Outcome is suicides among 15-24-year-old women** | | | **Model b: Outcome is suicides among 24-49-year-old women** | | |
| --- | --- | --- | --- | --- | --- | --- |
| **Parameters** | **Coefficient** | **95% CI** | | **Coefficient** | **95% CI** | |
|  |  | **Lower bound** | **Upper bound** |  | **Lower bound** | **Upper bound** |
| Suicides among males (for corresponding age group) | 0.25**** | 0.25 | 0.25 | 0.27**** | 0.26 | 0.28 |
| Autoregression (AR) lag |  |  |  |  |  |  |
| 3 | -0.51*** | -0.78 | -0.24 | NA | NA | NA |
| 4 | NA | NA | NA | 0.41*** | 0.16 | 0.67 |
| 6 | -0.42*** | -0.73 | -0.11 | NA | NA | NA |
| **Residuals statistics** | | | | | | |
| T- value of residual mean (against zero) | 0.06 | | | 0.13 | | |
| Standard deviation of residuals | 11.3 | | | 28.4 | | |

*p < 0.1; **p < 0.05, ***p < 0.01, ****p<0.001; two-tailed test

AR = AutoRegression; CI = confidence interval; NA = not applicable

**Supplement Figure S2: Time-series graph of ARIMA-derived expected (predicted) values of suicides among women 15-24 years (red) and 25-49 years of age (gray), United States, January 2018 to December 2023.**

**Supplement Figure S3. Time-series graph of ARIMA-derived residuals of suicides among women 25-49 years of age and pre-*Dobbs* 95% confidence (prediction) intervals of residuals (applied to full series), United States, January 2018 to December 2023. Residual outliers post-*Dobbs* (June 2022) are circled in red.**

**Supplement Table S2: Results from Ljung-Box Q test of residual autocorrelation in the series of monthly suicides among (i) 15-49-year-old women (Model a), (ii) 15-24-year-old women (Model b), and (iii) 25-49-year-old women (Model c).**

|  | Model a: ARIMA derived residuals of national series of monthly suicides among 15-49-year-old women | | | Model b: ARIMA derived residuals of national series of monthly suicides among 15-24-year-old women | | | Model c: ARIMA derived residuals of national series of monthly suicides among 25-49-year-old women | | |
| --- | --- | --- | --- | --- | --- | --- | --- | --- | --- |
| Lags | Autocorrelation Function | Standard Error | Ljung-Box Q Statistic | Autocorrelation Function | Standard Error | Ljung-Box Q Statistic | Autocorrelation Function | Standard Error | Ljung-Box Q Statistic |
| 1 | 0.05 | 0.15 | 0.1 | -0.05 | 0.15 | 0.1 | 0.15 | 0.14 | 1.1 |
| 2 | 0.09 | 0.15 | 0.5 | -0.07 | 0.15 | 0.4 | 0.06 | 0.15 | 1.3 |
| 3 | 0.08 | 0.15 | 0.8 | 0.04 | 0.15 | 0.5 | 0.07 | 0.15 | 1.6 |
| 4 | 0.09 | 0.16 | 1.2 | -0.14 | 0.15 | 1.5 | -0.06 | 0.15 | 1.8 |
| 5 | -0.09 | 0.16 | 1.6 | -0.18 | 0.16 | 3.1 | -0.05 | 0.15 | 1.9 |
| 6 | 0.06 | 0.16 | 1.8 | 0.06 | 0.16 | 3.3 | 0.18 | 0.15 | 3.8 |
| 7 | -0.17 | 0.16 | 3.3 | -0.12 | 0.16 | 4.2 | 0.17 | 0.15 | 5.5 |
| 8 | -0.2 | 0.16 | 5.5 | 0.01 | 0.16 | 4.2 | -0.03 | 0.16 | 5.5 |
| 9 | 0.04 | 0.17 | 5.6 | -0.02 | 0.16 | 4.2 | 0.14 | 0.16 | 6.8 |
| 10 | -0.1 | 0.17 | 6.1 | 0.07 | 0.16 | 4.5 | 0.11 | 0.16 | 7.5 |
| 11 | -0.15 | 0.17 | 7.5 | 0.02 | 0.16 | 4.5 | -0.07 | 0.16 | 7.9 |
| 12 | -0.16 | 0.17 | 9.1 | 0.18 | 0.16 | 6.6 | -0.01 | 0.16 | 7.9 |
